# Health Tract, No. 185

**Published:** 1865

**Authors:** 


### CORN BREAD.

A BUSHEL of corn contains as much nutriment as a bushel of wheat, and is five or six times less costly. But it is almost always spoiled in the Eastern States by being ground too fine. The most ignorant "contraband" in the South-West can make a most delicious bread in a few minutes out of corn-meal, pure water, and a little salt, baked on a hot hearthstone, or a heated hoe; this is the celebrated "hoe-cake" of the olden time. Very few persons in the East can make any kind of corn-bread without putting in soda, saleratus, or cream of tartar enough to physic an elephant; the necessity for these ingredients arises from the useless fineness of the meal, which makes it bake heavily. Chemical research has demonstrated that the most healthful and nutritious and strengthening particles of ground corn or wheat are found attached to the outer covering, which forms the "bran," and which, by some perversity, is segregated from both flour and meal. The same principle applies to the Irish potato, for there is more nutriment in the quarter of an inch attached to the skin than in the whole remainder. There is more of the element which forms our bones in the refuse bran of corn or wheat than in all the other parts together. From experiments recently made with cattle, it appears that there is a large amount of nutriment in the cobs of Indian corn; that if cobs and grain are ground together, cattle fare as well, thrive as well as if they were fed on the ground corn alone; and from the fact that those fed on the former gave about half as much more manure, it may be safely inferred that if the cob and corn were properly ground together, and eaten moderately coarse, but baked well and thoroughly, it would not only be a wholesome article of diet, but would have a good effect in remedying that "costive habit" which is almost inseparably connected with nearly every human ailment, which aggravates all of them, and the removal of which greatly ameliorates, if it does not promptly and permanently cure three fourths of our ordinary maladies, if combined with cleanliness, rest, and pure air.

MUSH AND MILK is a famous and much loved article of food, especially if the mush is slowly boiled for several hours; if it is then cooled, sliced, and fried, it makes a dish which a healthy and industrious man can eat with a relish every day in the year.

Indian corn coarsely broken, (called hominy,) soaked all night over or near the fire, and slowly boiled six or eight hours next day, makes a dish which may be eaten with salt, syrup, molasses, or milk, of which one scarcely ever tires.

